# DRG data for better decision-making in Croatia: planning for a greater use of same-day surgical admissions

**DOI:** 10.1186/1472-6963-15-S2-A4

**Published:** 2015-06-15

**Authors:** Karolina Kalanj, Karl Karol

**Affiliations:** 1 Karol Consulting, Zagreb, 10,000, Croatia

## Background

In 2006, Croatia implemented AR-DRGs with the aim of increasing hospital output transparency, incentivizing efficient resource use, and measuring hospital performance and quality. Despite the intentions of this initiative, up until very recently, the hospital payment system continued to be based on historical budgets, although hospitals have been reporting inpatient activity using the DRG classifications. Only now is transition to a DRG-based payment system being planned.

In 2013-2014, a Hospital Master Planning Project was undertaken to rationalize the hospital service delivery network and to improve efficiency. The Project also sought to create incentives to encourage the greater use of same-day inpatient services and improve the organization of the delivery of rehabilitation and palliative care services. The Project reported that the Croatian Health Insurance Fund (HZZO) does not have a strategy to encourage the utilization of same-day admissions. These services are primarily counted as part of the total DRG output for all admitted cases. Croatia uses AR-DRG version 5.2, which provides for 20 designated same-day DRGs.

In the case of same-day surgical cases, the HZZO pays hospitals through two parallel systems. The first is through DRGs, where there are only 2 are same-day surgical groups (C15B and C16B); the second is through the outpatient system, which has 17 same-day surgical case categories that, in terms of clinical activity, are replicated in the DRG system. The use of both systems and their uncoordinated pricing creates a situation in which hospitals do not have financial incentives to increase the use of same-day surgical procedures.

Recognizing that the increased use of same-day admissions would improve hospital efficiency, the Master Planning Project recommended that these admissions should be increased by 20% to 40% over a 3-year period. It appears, however, that these recommendations were made in the absence of a detailed analysis of the types of same-day procedures currently provided by hospitals. The Project did not recommend how hospital could increase their volume of same-day inpatient services, describe individual hospitals’ capacity to do so, or analyze hospital waiting lists.

## Materials and methods

We analyzed DRG activity data for Croatian Clinical Centres, because these institutions have both the greatest volume and the greatest potential to increase the number of same-day inpatient procedures, given appropriate incentives.

The analysis looked at HZZO DRG data from all of the Centres’ surgical cases in which patients were discharged either same-day or after one-night stay (one-day cases). The methodology was selected because it was probable that many of the one-day cases could have been treated on a same-day basis, given the appropriate financial incentives.

The data analysis also looked specifically at cataract surgery, which is included in both the DRG payment model (C15B) and the outpatient payment model. The analysis compared the incidence of same-day cataract surgery cases in other hospital systems.

## Results

According to HZZO’s DRG data, five Croatian Clinical Centres reported that between 22% and 33% (an average of 29%) of all surgical admissions were either same-day or one-day surgical cases (see Table 1). This is consistent with research from Italy reporting that one-day and same-day cases account for around 20% and 25% of all surgical cases [1].

**Table 1:** DRG patient data distribution, 2012

Clinical Centre	Number of admissions (NA)	Number of surgical admissions (SA)	Same-day and one-day surgical admissions (SOD)	SOD/SA %	Same-day cataract surgery cases as % of all cataract surgical cases
**Zagreb**	72,733	28,956	9,446	33%	11%

**Split**	51,030	19,249	5,465	28%	21%

**Osijek**	40,622	15,187	4,944	33%	0.3%

**Rijeka**	40,439	17,606	5,185	29%	25%

**Sisters of Mercy**	56,603	25,745	5,689	22%	9%

**Total/Average**	**261,427**	**106,743**	**30,729**	**29%**	**13%**

Source: 2012 DRG data from Croatian Health Insurance Fund (HZZO)

The number of cataract surgery cases performed by the Centres was comparatively low, compared to levels in other European Union (EU) countries. On average, 13% of the Croatian Clinical Centres’ cataract cases were undertaken on a same-day basis (see Table 1), compared to a 15-country EU average of 71% [2]. Other research indicates that low rates of same-day surgery for cataracts and other interventions result from disadvantageous payment models that do not provide incentives for same-day admissions [3].

## Conclusions

The Croatian hospital contracting model that is currently in use does not encourage the use of same-day admissions, and the payment system does not provide hospitals with incentives to discharge patients the same day. With the right incentives, however, we conclude that the Croatian Clinical Centres can significantly increase same-day admissions, which would improve their efficiency.

Croatian decision-makers should make appropriate use of the available DRG data to develop strategies to increase the use of same-day procedures, where clinically appropriate.

We also believe that, by not utilizing the available DRG data, it is likely that the Hospital Master Planning Project substantially underestimated the opportunity to increase same-day surgical cases.
